# Secondary syphilis mimicking tinea cruris in an HIV infected patient: a case report

**DOI:** 10.11604/pamj.2021.38.133.27922

**Published:** 2021-02-05

**Authors:** Cyntia Yuylana, Muji Iswanty, Idrianti Idrus Paturusi

**Affiliations:** 1 Department of Dermatology and Venereology, Faculty of Medicine, Hasanuddin University, Makassar, Indonesia

**Keywords:** Syphilis, mimicking, tinea cruris, case report

## Abstract

Syphilis is known as the great imitator with various clinical presentations which often lead to confusion and misdiagnosis. A 28-year-old male presented with non-pruritic and painless erythematous patches around the anus and scrotum. Initial differential diagnosis with tinea cruris. Fungal examination was negative. Serological tests for syphilis were positive and anti-HIV screening was reactive. A diagnosis of secondary syphilis was established and the patient was given intramuscular injection of 2.4 million unit of benzathine penicillin. The skin lesions improved significantly 1 week after treatment, confirming a diagnosis of secondary syphilis with HIV. Annular skin lesions in secondary syphilis are uncommon and often misleading. This case emphasizes the importance of considering secondary syphilis in the differential diagnosis of annular lesions.

## Introduction

Syphilis (lues, the great mimic, the great masquerader, the great imitator, and neapolitan disease) is a sexually transmitted infection caused by the *Treponema pallidum* subspecies *pallidum* [1-3]. The clinical course of syphilis is divided into 3 stages: primary, secondary, and tertiary. The clinical presentation of secondary syphilis varies widely and may resemble that of other diseases [4]. The diverse manifestations of secondary syphilis earn it the name “the great imitator” and often leads to diagnostic delay and mistreatment [5]. Here, we report an atypical presentation of secondary syphilis that mimicked tinea cruris which emphasized the need to increase awareness to unusual clinical presentation of syphilis.

## Patient and observation

A 28-year-old male presented with a 6-month history of an erythematous patch around the anus to the Department of Dermatology and Venereology of Gatot Soebroto Army Hospital, Jakarta, Indonesia. Two months ago, erythematous macules also appeared on the scrotum. The patient did not complain of itching or pain. Initially, the patient was diagnosed with dermatophytosis and was treated with topical antifungal agents for four weeks without any improvement. The patient was not married and admitted a history of multiple occasions of unprotected same-sex sexual intercourse in the past one year.

**Figure 1:**
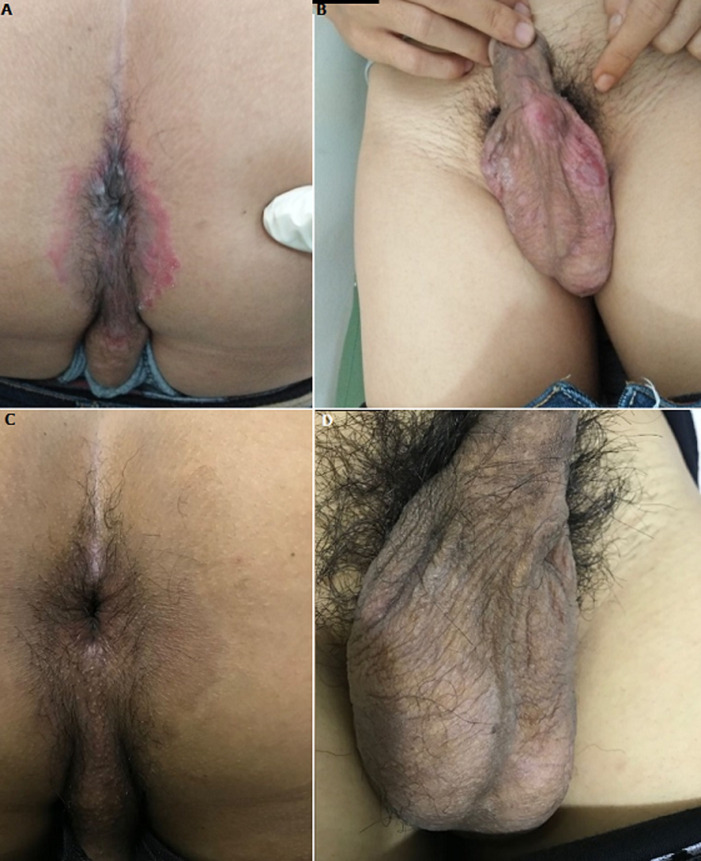
(A and B) annular erythematous scaly lesions were visible on the anus and scrotum; (C, D) one week after therapy

The results of physical examination were within normal limit. Dermatological examination revealed plaques, annular erythematous, defined borders, with a fine scale overlying on the perianal region (Figure 1 A) and erythematous macules and erosions on the scrotum (Figure 1 B). The results of venereal disease research laboratory test (VDRL) and *treponema pallidum* particle agglutination assay (TPHA) examinations were positive (positive at 1: 256 and 1: 10240). Anti-HIV screening was reactive with a CD4 count of 569 cells/uL. Skin scraping examination for fungal infection came out negative. Based on the patient´s history of unprotected sexual behavior, clinical manifestations and serological findings, the patient was diagnosed with secondary syphilis and HIV infection. The patient was treated with a single intramuscular injection of 2.4 million unit of benzathine penicillin G. After 1 week of treatment, the skin lesions markedly improved and the patient was advised to return for follow-up after three months (Figure 1C and Figure 1D).

## Discussion

Men accounted for 91% of all cases of primary and secondary syphilis in USA [6]. During 2013-2014, the primary and secondary syphilis rate increased 14.4% in men [1]. Rates of primary and secondary syphilis were highest in people aged 20 to 29 years old [1, 7]. Lesions in secondary syphilis may occur 6 weeks to 6 months after infection [1, 8]. The cutaneous manifestations of secondary syphilis are diverse. The classic clinical manifestation of secondary syphilis is a diffuse maculopapular rash that often, but not always, involves the palms and soles, and scrotum. However, the rash can also be papular, annular, or pustular, and can have fine overlying scale [5]. Rash in secondary syphilis may present as reddish-brown, copper or slightly bluish (50-70%) maculopapular lesions, but can also be in the form of papules (12%), macules (10%), and annular papules (6-14%) [2].

Annular configuration is one of the atypical skin manifestations of secondary syphilis. Papular lesions with annular morphology on the surface of the palms have been observed as a manifestation of secondary syphilis in patients with a previous history of syphilis. In another report, lesions developed in the form of symmetrical, erythematous, annular and arcuate plaques that were more extensive on the scalp, lower body, perioral, perianal and genital areas. Annular lesions may vary from thin, slightly raised lesions with scaly borders to thicker verrucous plaques that can be purplish in color [8]. Lesions in syphilis usually are not pruritic [1, 8], although in a study pruritus was reported in about 40% of patients [1]. In this case, the erythematous annular scaly plaque with clear border mimicked tinea cruris. In this patient, the possibility of tinea cruris could be excluded because KOH examination did not find hyphae or arthrospores. This is in accordance with the literature which states that based on the location of the anatomy and clinical features, one of the differential diagnosis of secondary annular syphilis is dermatophytosis [9].

Based on a research in Atlanta, Los Angeles and New York City, 45% of men with syphilis also had HIV. Genito-ulcerative diseases (such as syphilis) cause damage to the skin barrier and thereby increase the risk of HIV transmission [6]. In patients with syphilis infection, the risk of HIV transmission is increased due to the release of the virus in the wound or genital ulcer. Thus, HIV screening is recommended in patients with syphilis [10]. In the majority of HIV-infected syphilis patients, syphilis can be diagnosed by serologic testing [9].

Syphilis therapy in HIV positive and negative patients are indifferent. According to the WHO guideline, the recommended treatment for patients with primary and secondary syphilis is intramuscular injection of 2.4 million units of benzathine penicillin G. The alternative regimens are procaine benzylpenicillin 0.6 million units/day, doxycycline, azithromycin and ceftriaxone [1-5, 7, 8]. In patients with no HIV infection, CDC recommends follow-up at intervals of 6 months in which a four-fold decrease in titer is expected. However, in HIV-infected persons, the recommended follow-up are at 3, 6, 9, 12, and 24 months [1, 3, 4].

## Conclusion

This case emphasizes the importance of considering secondary syphilis in the differential diagnosis of annular lesions.
